# Antimicrobial Potential of Curcumin: Therapeutic Potential and Challenges to Clinical Applications

**DOI:** 10.3390/antibiotics11030322

**Published:** 2022-02-28

**Authors:** Yaseen Hussain, Waqas Alam, Hammad Ullah, Marco Dacrema, Maria Daglia, Haroon Khan, Carla Renata Arciola

**Affiliations:** 1 College of Pharmaceutical Sciences, Soochow University, Suzhou 215123, China; pharmycc@gmail.com; 2 Department of Pharmacy, Abdul Wali Khan University Mardan, Mardan 23200, Pakistan; waqasalamyousafzai@gmail.com; 3 Department of Pharmacy, University of Naples Federico II, 80131 Naples, Italy; hammadullah@unina.it (H.U.); marco.dacrema@unina.it (M.D.); 4 International Research Center for Food Nutrition and Safety, Jiangsu University, Zhenjiang 212013, China; 5 Laboratorio di Patologia delle Infezioni Associate all’Impianto, IRCCS Istituto Ortopedico Rizzoli, Via di Barbiano 1/10, 40136 Bologna, Italy; carlarenata.arciola@ior.it; 6 Laboratorio di Immunoreumatologia e Rigenerazione Tissutale, IRCCS Istituto Ortopedico Rizzoli, Via di Barbiano 1/10, 40136 Bologna, Italy; 7 Department of Experimental, Diagnostic and Specialty Medicine, University of Bologna, Via San Giacomo 14, 40136 Bologna, Italy

**Keywords:** curcumin, antimicrobial potential, clinical challenges, nanocurcumin, nanoparticles, orthopedics

## Abstract

Curcumin is a bioactive compound that is extracted from *Curcuma longa* and that is known for its antimicrobial properties. Curcuminoids are the main constituents of curcumin that exhibit antioxidant properties. It has a broad spectrum of antibacterial actions against a wide range of bacteria, even those resistant to antibiotics. Curcumin has been shown to be effective against the microorganisms that are responsible for surgical infections and implant-related bone infections, primarily *Staphylococcus aureus* and *Escherichia coli*. The efficacy of curcumin against *Helicobacter pylori* and *Mycobacterium tuberculosis*, alone or in combination with other classic antibiotics, is one of its most promising antibacterial effects. Curcumin is known to have antifungal action against numerous fungi that are responsible for a variety of infections, including dermatophytosis. Candidemia and candidiasis caused by *Candida* species have also been reported to be treated using curcumin. Life-threatening diseases and infections caused by viruses can be counteracted by curcumin, recognizing its antiviral potential. In combination therapy with other phytochemicals, curcumin shows synergistic effects, and this approach appears to be suitable for the eradication of antibiotic-resistant microbes and promising for achieving co-loaded antimicrobial pro-regenerative coatings for orthopedic implant biomaterials. Poor water solubility, low bioavailability, and rapid degradation are the main disadvantages of curcumin. The use of nanotechnologies for the delivery of curcumin could increase the prospects for its clinical application, mainly in orthopedics and other surgical scenarios. Curcumin-loaded nanoparticles revealed antimicrobial properties against *S. aureus* in periprosthetic joint infections.

## 1. Introduction

The prevalence of antibiotic resistance towards microorganisms is progressively increasing around the globe, and resistance to antibacterial drugs is among the primary causes of therapeutic failure. To cope with this situation, an effective, safe, and economical natural product or phytochemical is required. From prehistoric times, therapies obtained from plants and their phytochemicals have been essential for health maintenance. Traditional medicines have been reported to have a significant influence on the treatment of pathogens by the regulation of diverse physiological processes in countless research studies based on experimental models and preclinical findings [1]. Curcumin is a bioactive curcuminoid polyphenol that is isolated from the rhizomes of *Curcuma longa*. Chemically, curcumin is 1,7-bis(4-hydroxy-3-methoxyphenyl)-1,6-heptadiene-3,5-dione. It is also termed as diferuloyl methane [2]. *Curcuma longa* is commonly known as turmeric, which belongs to the *Zingiberaceae* family. Turmeric is found abundantly and naturally in tropical areas, on the Indian subcontinent, and in South Asia. Turmeric is dark yellow in color due to the presence of a wide variety of polyphenolic curcuminoids. Curcuminoids such as curcumin, bisdemethoxycurcumin, and dimethoxy-curcumin are found in *Curcuma longa* [3].

Turmeric has been used in the Ayurvedic medicine system for the management of various medical disorders such as jaundice, skin infections, wounds healing, flatulence, sprains, arthritis, and stomach disturbances since ancient times. Curcumin has also been established as an anti-asthmatic, antiarthritic, anti-inflammatory, antioxidant, antimicrobial, cardio-protective, and immuno-modulatory agent [4]. Curcumin targets several signaling molecules while illustrating cellular activity, supporting its numerous health benefits. Curcumin supplements have been found to have potential nephroprotective, analgesic effects and to be useful in the management of metabolic syndromes because of its antioxidant effects [5,6].

Although curcumin has a broad spectrum of pharmacological properties, a critical challenge towards desirable therapeutic applications is its poor bioavailability, which is due to its poor intestinal absorption, hydrophobic character, and rapid metabolism. Its systemic bioavailability is very low after oral administration. However, studies have found that a small amount of systemically available curcumin has a marked therapeutic effect. Different agents were analyzed to better determine the bioavailability of curcumin [7]. Curcumin is considered as a potential agent for the development of novel natural products, including nanocrystals and micro-particles, to improve its stability versus the identified factors, and to manipulate bioactivities [8,9]. The antimicrobial mechanistic pathway of curcumin usually includes interference from fundamental cellular division and the induction of the protein-filamenting temperature-sensitive mutant Z (FtsZ). The cytoskeleton of bacteria is essential for development and cell division, whereas the FtsZ protein is associated with microbial cell replication and is the first protein that emerges at the imminent division site [10,11].

Researchers have reported that the methoxy and hydroxy derivatives of curcumin are specifically related to its antibacterial efficacy [12,13]. Although many some reviews on the use of curcumin and its analogues that are associated with its antimicrobial and anti-parasitic properties in specific applications (i.e., wound healing, periodontal diseases, tuberculosis, *Helicobacter pylori* infection) have been published in recent decades [14,15,16], there is a need for a comprehensive literature review that includes both the studies that have been performed on the antimicrobial activity of curcumin and those reporting the technological approaches in order to have a vision of what has been achieved so far and to direct future studies.

Thus, the aim of this review is to design an updated literature review on the antimicrobial effect of curcumin (antibacterial, antifungal, and antiviral activities) in different formulations to improve its bioavailability, highlighting the possible role of curcumin in orthopedics due to its antibacterial and osteogenic effects as well as limitations and the future insights for the management of infectious diseases in clinical practice.

## 2. Methodology

**Figure 1:**
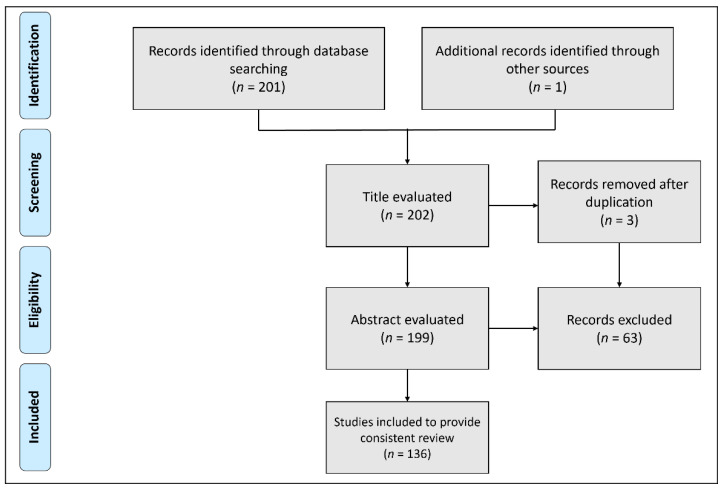
PRISMA flow diagram showing the study selection process.

This research covered all studies addressing the antimicrobial potential of curcumin and nanotechnological approaches to improve the bioavailability of curcumin. For study selection, we used up-to-date databases, including Web of Science, Pubmed, Scopus, and Google Scholar. The keywords used in our search were “turmeric”, “*Curcuma longa*”, “curcumin”, “curcuminoids”, “infections”, “antimicrobial activities”, and “nanoparticles”. The only criterion for selecting articles was “studies reported in English, because of language barrier”. The results returned 201 papers and 1 book chapter published up to the year 2022. Of these, 136 articles were selected, summarized, and critically discussed to provide a consistent review. The main reasons for the exclusion of articles were the fact that they were written in a language other than English or because they were title duplications. Figure 1 illustrates the PRISMA flow diagram for the study selection process.

## 3. Antibacterial Effect

### 3.1. In Vitro Assays

Curcumin inhibits the growth of both Gram-negative and Gram-positive bacteria. [17,18]. According to the findings of an in vitro research study, the minimum inhibitory concentration (MIC) of curcumin needed to suppress the growth of methicillin-resistant *Staphylococcus aureus* (MRSA) species is 125–250 g/mL [19]. Curcumin, one of the main components of turmeric, inhibited the growth of all *Helicobacter pylori* species in patients with gastrointestinal disorders in vitro [20]. Negi and his co-authors evaluated the antibacterial effect of curcumin via the pour plate method. Different curcumin fractions were investigated against *Pseudomonas aeruginosa*, *Staphylococcus aureus*, *Bacillus subtilis*, *Bacillus cereus*, *Escherichia coli,* and *Bacillus coagulans*. They determined that curcumin has a significant antimicrobial effect against all of the studied strains of bacteria [21]. In an in vitro analysis, researchers evaluated the antibacterial effect of novel curcumin compounds (indium curcumin, diacetyl-curcumin, and indium diacetyl-curcumin) versus curcumin.

Different antimicrobial strains such as *Escherichia coli, Pseudomonas aeruginosa, Staphylococcus epidermidis,* and *Staphylococcus aureus* were assayed. They reported that curcumin showed potential antibacterial activity against all of the tested species, indium diacetyl-curcumin revealed an antibacterial effect against *Staphylococcus epidermidis* and *Staphylococcus aureus,* diacetyl-curcumin had no antibacterial effect on any species, whereas it was reported that indium curcumin demonstrated significant antibacterial potential against all of the tested strains. They also determined the MIC of all of the compounds against each of the antibacterial species and found that the MIC of curcumin against *Staphylococcus aureus* and *Staphylococcus epidermidis* was 187.5 µg/mL and 46.9 µg/mL, respectively. The MIC for indium curcumin was determined to be slightly less than that of the other two species above, which were 93.8 µg/mL and 23.4 µg/mL, respectively. Thus, their analysis established that indium curcumin has a higher antibacterial effect compared to curcumin [22].

Izui et al. assayed the antimicrobial activity of different concentrations of curcumin against periodontopathic microbes (*Streptococcus gordonii, Prevotella intermedia, Porphyromonas gingivalis, Treponema denticola, Fusobacterium nucleatum, and Aggregatibacter actinomycetemcomitan)* by means of spectro-fluoro-photometric analysis. From their investigations, they concluded that different concentrations of curcumin retarded the development of *T. denticola, P. intermedia, P. gingivalis,* and *F. nucleatum* [23]. Another in vitro investigation reported the antimicrobial properties of curcumin in the species comprising the typical deepest periodontal microbes: *Actinomyces viscosus, Lactobacillus casei, and Streptococcus mutans,* as well as in the main root canal bacterial species: *Enterococcus faecalis, Prevotella intermedia and Porphyromonas gingivalis.* Their findings showed that, excluding *E. faecalis*, curcumin demonstrated antimicrobial activity versus all of the tested species [24]. 

Basniwal et al. reported the antimicrobial properties of curcumin nanoparticles versus different microbial strains such as *Escherichia coli, Staphylococcus aureus, Pseudomonas aeruginosa, Penicillium notatum*, *Aspergillus niger, and Bacillus subtilis.* Curcumin nanoparticles that were 2–40 nm in size were designed using the wet milling method. These nanocurcumin particles were observed to be readily soluble in freshwater without the use of surfactants. These findings showed that reducing the size of curcumin particles to the nanoscale significantly enhanced their solubility in water and their antibacterial properties. They reported that Gram-positive bacteria were more susceptible to nanocurcumin compared to Gram-negative bacteria [25]. Core–shell copper oxide–curcumin nanocomposite technologies for antibiotics were synthesized and implemented by Khorsandi et al. for use against *Shigella dysenteriae, Streptococcus pneumonia, Escherichia coli,* and *Staphylococcus aureus* [26].

They also reported that in sterile distilled water, the antibiotic nanoparticles were extremely soluble. Furthermore, it was discovered that this antimicrobial research revealed that antibiotic core–shell nanoparticles and nanocomposites significantly outperformed amoxicillin (standard antibiotic) [27]. Mody and his co-authors evaluated the anti-*Clostridium* effect of isolated bioactive derivatives of curcumin. Approximately 27 strains of *Clostridium difficile* (both spore and toxins forming) were assessed against the active compounds of *Curcuma longa* (curcumin, bis-demethoxy-curcumin, and demethoxy-curcumin). They revealed that curcumin markedly prevented *Clostridium difficile* culture growth at varying concentrations of between 6 and 33 μg/mL. Moreover, they also evaluated the action of curcumin on normal intestinal flora, such as *Lactobacillus*, *Bifidobacterium,* and *Bacteroides,* and concluded that curcumin has no antibacterial effect against normal flora [28]. A summary of the different experimental studies that have been performed to determine the antibacterial effect of curcumin is presented in Table 1.

### 3.2. In Vivo Assays

A lot of in vitro investigations have shown that curcumin possesses antimicrobial activity towards microbes such as and Gram-positive and Gram-negative bacteria [19,30,36,37]. Curcumin has a wide range of mechanistic pathways that are responsible for these antimicrobial actions. These pathways may include DNA replication inhibition, the modifications in plasmid gene expression, cell membrane deterioration, and motility reductions. De and his co-researchers evaluated the antimicrobial activity of curcumin in an animal model. They investigated curcumin’s antibacterial activity in *H. pylori* infected mice as well as its effectiveness in minimizing the gastric injury caused by infection histopathologically. They found that curcumin proved to be extremely influential in both eradicating *H. pylori* from experimental animals and in restoring *H. pylori*-induced stomach injury, indicating that it has enormous protective effects against *H. pylori* [20].

Wang and his co-researcher reported the antibacterial activity of curcumin in a mice model and established that curcumin therapy protected the mice against *S. aureus*-induced strains of pneumonia, such as MRSA. Curcumin inhibits the pore-forming behavior of α-hemolysin (a major pathogenic variable for the progression and pathogenesis of *S. aureus* pneumonia) via an innovative pathway, paving the ground for the production of new and improved antimicrobial drugs against *S. aureus* infection. They further concluded that curcumin supplementation substantially lowered the α-hemolysin-mediated harm imparted to the alveolocytes (co-cultured with *S. aureus*) [34]. Curcumin-loaded Nisin-based poly (L-lactic acid) nanoparticles were fabricated and evaluated in wound infections in vivo. The results showed a significant reduction in the wound size in mice, suggesting the effective antibacterial potential of curcumin in burn wound infections [38]. Similarly, an in vivo evaluation of curcumin on multidrug-resistant *E. coli* isolates showed that curcumin had potential to be an effective antibacterial [39].

### 3.3. Antibacterial Resistance

Curcumin has been reported to be a promising breaker of antimicrobial resistance, as curcumin has the potential to restore the effectiveness of failed antimicrobials by lowering their MICs. Researchers investigated the resistance of a multi-drug resistant strain of *Mycobacterium abscessus* (isolated from a 66-year-old tuberculosis patient) against curcumin. In 2018, Marini treated resistant strains of *M. abscessus* with a combination therapy with different concentration of curcumin with clarithromycin, linezolid, ciprofloxacin, and amikacin. Curcumin slightly decreased the pathogenicity (degree or state of being pathogenic) at 1/8 × MIC, while at 4 × MIC, it totally blocked biofilms from 4 to 8 days. Additive curcumin and amikacin therapy caused a significant decline in the viable cell count as well as a decrease in the microbial colonies. While curcumin was the main agent, the most notable feature observed was the biofilm destruction on the 4th and 8th days. These results backed up past evidence that curcumin can help break resistance to antibiotics. Curcumin, alone or in combination with antibiotics, may help to establish a new approach for combating the pathogenicity and drug resistance of *M. abscessus* [40]. Methicillin-resistant *Staphylococcus aureus*-related hospital infections are a global public health issue. In combination with other antibiotics, curcumin is effective against such resistance. Due to its low systemic bioavailability, curcumin was formulated in the form of graphene oxide nanoparticles and was targeted for antibiotic activity. The fabricated curcumin nanoparticles showed low toxicity and effective anti-bacterial activity against antibiotic-resistant infection at concentrations of less than 2 µg/mL [41].

## 4. Antifungal Effect

Due to the widespread conventional utilization of turmeric in edible items, several studies have been conducted to investigate curcumin and turmeric in the context of preventing fungal contamination and infections. Martin and his co-authors investigated the protective effect of curcumin versus 23 fungal species in vitro. Fungicidal vulnerability was determined using a CLSI-approved broth micro-dilution technique. Curcumin was quite effective against isolated strains of *Paracoccidioides brasiliensis*, but it had no effect on *Aspergillus* strains. The adherence of *Candida* species was more efficiently inhibited by curcumin compared to fluconazole, especially in the buccal mucosal strains that were isolated from patient suffering from AIDS, proving that curcumin is indeed a potential natural compound that deserves more research into its pharmacological activity in immunosuppressed individuals. Thus, the researchers concluded that curcumin is an effective antifungal agent for the management of *P. brasiliensis* infection compared to fluconazole [42].

**Figure 2:**
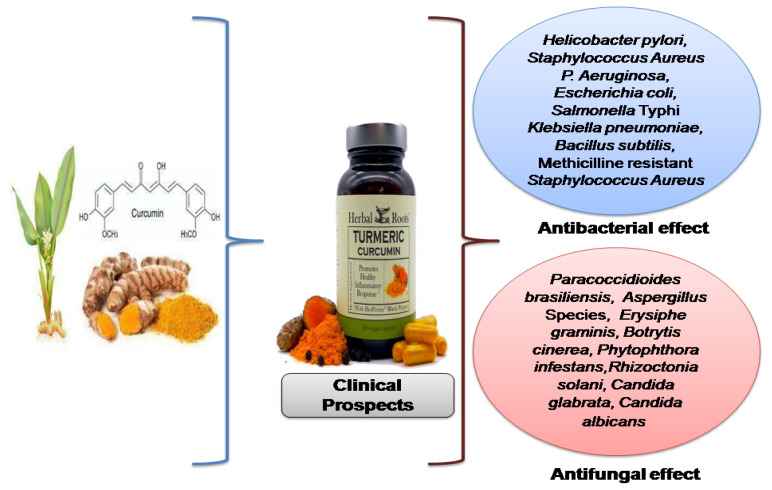
Anti-microbial spectrum of curcumin.

Neelofar and her co-researchers performed an in vitro assay on curcumin outcomes on proteinase secretions, H^+^ extrusion by ATPase, sterol content, and the growth of *Candida glabrata* and *Candida albicans* versus fluconazole treatment. It was noted that curcumin reduced H+ extrusion from 28% to 18% when glucose was available and from 42% to 32% when glucose was not available. In *C. albicans,* the proteinase secretions were reduced by fluconazole and curcumin to about 53% and 49%, respectively, whereas the reduction percentages in *C. glabrata* were about 46% and 39%, respectively. Curcumin has been found to be efficient against both the reference and clinical strains that have been study up until now, but in different ways than fluconazole. Curcumin’s antifungal efficacy may be attributed to changes in the biosynthesis of ergosterol, proteinase secretions, and ATPase activity that occur at membrane level 35. Researchers have also established the antifungal activity of *Curcuma longa* in different fungal species such as *Erysiphe graminis, Botrytis cinerea, Phytophthora infestans,* and *Rhizoctonia solani* [43]. Research findings have proven that concomitant therapy using vitamin C and curcumin has marked antifungal effects despite versus therapy with either on their own [44]. These combination therapies have shown that the combination of various fungicide products and curcumin may generate a synergism that increases the effectiveness of the antifungal approaches that are currently available. Thus, this finding provides future insights for the use of combination therapy in clinical practice. A graphical presentation of the antimicrobial effect of curcumin is provided in Figure 2.

## 5. Antiviral Effect

**Figure 3:**
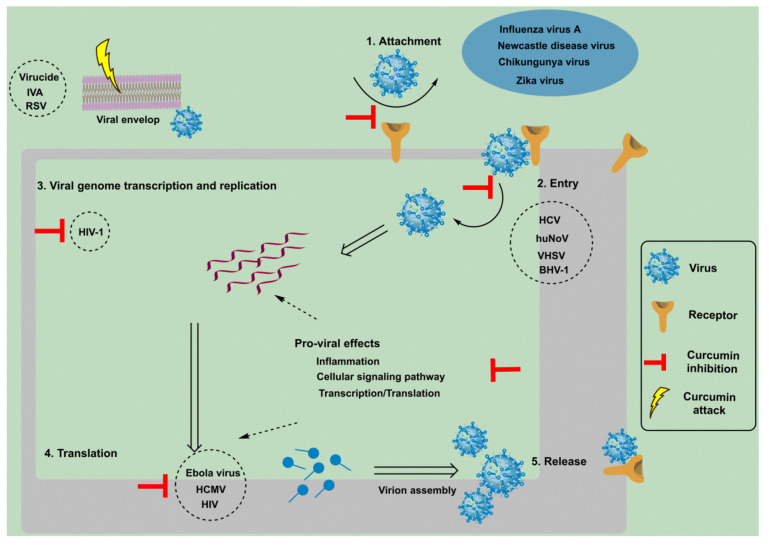
Antiviral mechanism of curcumin in the host cell. Chemotherapeutic action is attracted to certain crucial stages in the viral life cycle. These steps include the attachment of the virion to its cellular receptor, its consecutive entry, followed by the viral genome transcription and replication step, and then its translation, virion assembly, and finally release. Curcumin inhibits the action of viral envelope proteins, preventing viral attachment and entry. In addition, certain signaling pathways, inflammation, and translation/transcription machineries are modulated by curcumin that then becomes block viral replication. Apart from this, curcumin disrupts the integrity of the viral envelope and thus acts as a virucidal agent. A few of the viruses against which curcumin has shown a versatile antiviral effect are shown in the circles.

Curcumin appears to be a potent antiviral agent against various viruses, including feline infectious peritonitis virus, respiratory syncytial virus (RSV), para influenza virus type 3, herpes simplex virus (HSV), vesicular stomatitis virus, and flock house virus, as well as others [45]. Combating viral diseases, particularly those triggered by evolving viruses, have always been a dilemma. Curcumin’s antiviral properties emerge from its potential to modulate a variety of the molecular targets that are involved in cellular events such as transcription regulation and the activation of cellular signaling pathways such as the apoptosis and inflammation pathways through intermolecular interaction [46]. The mechanism and events adopted by curcumin to initiate antiviral activity are shown in Figure 3.

Viruses grow on the surface of cell membrane through attachment—this is considered as the initial event. According to a recent study, infections that are induced by arthropod-borne viruses such as Chikungunya and Zika are able to be prevented by curcumin by preventing the virus from attaching to the cell surface [47]. Curcumin enhanced lipid raft development in Madin–Derby bovine kidney (MDBK) cells, affecting the entry stage of bovine herpes virus type 1, reducing their total viral yield in a dose-dependent manner [48]. In addition, curcumin inhibited the entry of all of the major hepatitis C virus genotypes in a consistent manner. In membrane fluidity studies, viral entry was hampered by two events: viral binding and membrane fluidity. Curcumin caused fluidity modifications in the viral envelop that resulted in fusion [49]. Despite this, a non-enveloped virus, human norovirus (HuNoV), showed the influence of curcumin at an early stage of viral infection. At different doses and intervals, curcumin therapy has been shown to have antiviral effects such as viral entry rather than HuNoV RNA replication [50]. This is because viral entry requires the initial interaction of the surface protein on virus with the host membrane receptor. Curcumin can affect viral entry in the case of HuNov by preventing the action of the viral surface proteins, which does not have a lipid bilayer envelop structure as a target. Curcumin has also been studied in the context of photodynamic therapy at a concentration of 5 µM, where the compound was “activated” by exposure to particular light wavelengths, resulting in the release of reactive oxygen species. When photodynamically triggered by blue light radiation, curcumin has a greater effect on MuNoV titers than curcumin or blue light alone [51]. Photodynamic activity (PDAC) was also tested against noroviruses in another study that used feline calicivirus and MuNoV as HuNoV surrogates. PDAC treatment with up to 13.6 M curcumin decreased the 50% tissue culture infective dose (TCID50) of both feline caliciviruses (FCV) and murine noroviruses (MuNoV) but proved to be more successful against FCV [52]. Given that curcumin is now regarded as being safe for use in the food industry, the technique of using PDAC against noroviruses in oysters provides a potentially easy solution to fight norovirus accumulation.

Curcumin has shown its antiviral effects against Zika virus (ZIKV) by preventing cell attachment. Only the cells that had been treated before or after infection decreased Zika virus recovery in multiple time-of-addition assays, meaning that curcumin predominantly works against ZIKV at cell entry/attachment and not during later stages of infection [47]. Anti-Zika virus activity was evaluated for curcumin. The plaque assay in Vero E6 cells indicated that curcumin exhibited an IC_50_ value against several Zika virus strains. The cell attachment interference caused by curcumin for Zika virus was confirmed through time-of-addition analysis [53]. These findings show the antiviral potential of curcumin against Zika virus. Similarly when curcumin was incubated at a concentration of 4.5 µM with vesicular stomatitis virus, the results indicated that curcumin inhibited viral infection [54]. In addition, the antiviral activity of curcumin was evaluated for enveloped viruses targeting transmissible gastroenteritis viruses. Curcumin was incubated with the virus at a concentration of 20 µM and successfully reduced viral yield as well as viral absorption [55]. In another study, curcumin was incubated with porcine reproductive and respiratory syndrome virus at a concentration of 15 µM, reducing the infection. Internalization and membrane fusion mechanisms were involved. In viral hemorrhagic septicemia viruses, curcumin effectively inhibited viral replication with improved cell viability [56].

Curcumin’s antiviral effects have been studied extensively, with the majority of studies focusing on its effectiveness against HIV. Curcumin does have an effect on HIV function at various levels of the virus’s life cycle. Curcumin can directly interact with the protein catalytic core and can inhibit viral integration. Curcumin’s diketone moiety is thought to be responsible for its decay at physiological pH curcumin A, a synthetic curcumin analogue without the diketone moiety and that was tested for antiHIV-1 properties in a research study. Curcumin A had better stability in phosphate buffer and serum in vitro than curcumin did but had comparable stability in a tissue culture medium [57]. The effectiveness of tetrahydro–curcumin—a colorless curcumin metabolite and a possible topical vaginal microbicide used for prophylactic purposes to combat HIV infection—was evaluated. Tetrahydro–curcumin inhibited HIV-1 more effectively than the drug alone in a reporter cell line [58]. Apart from this, when evaluating curcumin against HIV virus, a research group tested the efficacy of curcumin against dengue virus. Four strains of dengue virus were evaluated, in which curcumin effectively reduced plaque formation with minimal toxicity. The mechanistic approach that was observed was took place at the cellular level rather than on viral functions [53,59]. Curcumin, bisdemethoxycurcumin, and its three synthesized analogues were also tested for their anti-dengue virus properties. Curcumin and its four analogues moderately blocked viral protease function in an in vitro activity assay [60]. Curcumin also blocked the Chikungunya virus entry and ultimately blocked it from causing infection in an in vitro study [54]. Chikungunya infection being inhibited by curcumin and its nanoparticles has been reported in many studies [61].

Curcumin appears to work at various levels of the virus life cycle and is considered to be a potent inhibiter of influenza-A viruses. When incubated with an influenza-A virus, curcumin successfully reduced viral infectivity, which was presumably due to curcumin’s ability to interfere with the haemagglutinin activity of the associated virus [62,63]. The replication of influenza-A viruses is modulated by the NF-κB signaling pathway, and curcumin effectively inhibits that signaling pathway, demonstrating that it has antiviral potential against influenza-A virus [64]. Curcumin can combat the diseases and infections that are associated with influenza-A viruses. This statement was justified in a research study that demonstrated that mice that had been infected with influenza-A virus in in a lung tissue model were more likely to survive and less likely to lose body weight after being treated with curcumin [65]. The enone functional groups in curcumin pose partial influenza-A virus activity. The sulfhydryl groups of the viral surface proteins form Michael adducts with these enone groups, contributing to a conjugate formation between curcumin and the viral surface protein that ultimately results in viral function interference [66]. Apart from these aspects, curcumin was also evaluated for influenza-A virus at the molecular level, and this evaluation indicated that curcumin successfully reduced the mRNA levels in the M gene of influenza-A virus cells that had been infected with the maximum nontoxic doses [67].

Vero cells are kidney epithelial cells (non-human); when curcumin was used to target these cells, it successfully blocked the replication of enterovirus-71. Curcumin was also evaluated in intestinal epithelial cells. The results of the study suggested that curcumin significantly reduced genome replication along with the protein expression of enterovirus-71 [68,69]. In terms of curcumin’s antiviral effect on SARS-CoV-2, the causative agent in the worldwide COVID-19 pandemic, it has been hypothesized that curcumin could inhibit SARS-CoV-2 replication. It has been shown that curcumin suppress the replication of SARS-CoV-1, the corona virus that triggered the 2003 outbreak [70]. Curcumin has also been shown to be effective in inhibiting SARS-CoV-2 replication through interaction with the spike glycoprotein in many molecular docking studies. In addition, other mechanisms include the inhibition of the main viral protease, the nonstructural proteins of the virus, and angiotensin-converting enzyme-2 [71,72]. Curcumin combined with polymers was evaluated against HSV-2 in a mice model. When using nanoparticles, the authors noted that it is difficult to decide whether an optimal dose has been met while controlling the dosage with a combination of crude extracts. Increasing the solubility makes it easier, resulting in an effective outcome against HSV-2 replication being observed.

**Figure 4:**
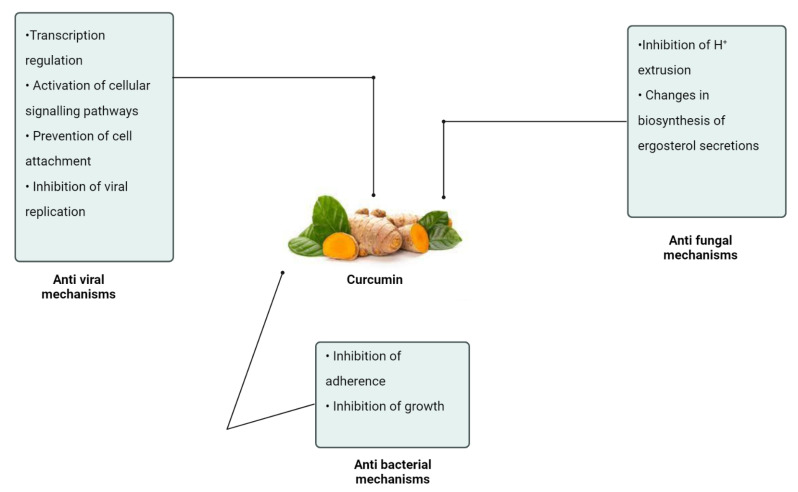
Antimicrobial mechanisms of curcumin.

The major antimicrobial mechanisms induced by curcumin are depicted in Figure 4.

## 6. Synergistic Effects

In combination therapy, natural products offer their purported medicinal effects. In contrast, using nanoparticles as therapeutic agents for oral use can help to overcome certain drawbacks and to provide benefits over selective chemotherapy in the form of low toxicity and the stimulation of antibiotic resistance [73]. However, combination therapy also helps to overcome some of the associated barriers. In this regard, curcumin and quercetin were co-delivered to evaluate its antibacterial potential. The results suggested that the co-delivery of curcumin and quercetin showed antimicrobial activity against MRSA at lower concentrations compared to their individual administration, and the resultant effect was in the form of synergy [74]. Bacterial growth can also be controlled using a new approach—photodynamic inactivation. *Staphylococcus aureus* growth was inhibited by the combination therapy of curcumin and hypocrellin B, where the photodynamic efficacy of hypocrellin B was potentiated by curcumin [75].

Combination therapies can also be used to overcome the resistance crisis. When hybridized with octa-arginine—a cell penetrating peptide, curcumin showed good antibacterial action. The result was in a synergic form with a bactericidal effect and occurred through curcumin targeting the bacterial cell membrane [76]. Similarly, another small peptide—bacteriocins—has demonstrated efficient anti-bacterial activity; however, due to resistance, it does not provide the desired therapeutic output. Curcumin was co-administered with bacteriocins against *Staphylococcus epidermidis* and *E. coli*. Curcumin potentiated the antibacterial activity of bacteriocins, showing that it can be used in biomedical applications in combination with curcumin [77]. Curcumin was also co-delivered with suberoylanilide hydroxamic acid in order to improve its water solubility and efficacy. The results showed that such a synergistic combination displayed stronger antibacterial action for curcumin than suberoylanilide hydroxamic acid and pure curcumin alone [78].

Drug-resistant strains of *Staphylococcus aureus* are to the cause of infection-related mortality. Many antibiotics have been rendered inactive due to the advent of drug resistance. Recurrent latent infections are caused by the low penetration and retention of antibiotics in mammalian cells. Individually, curcumin and berberine were shown to be less effective due to low penetration and hydrophobicity. The co-delivery of both once again showed no remarkable antimicrobial synergistic effect. However, the loading of both into liposomes displayed a significant synergistic effect against MRSA [79]. Similarly, curcumin in combination with xylitol was evaluated for its antibacterial and antifungal activities. The combination effect was in the form of synergistic effect against Staphylococcus aureus, *P. aeruginosa,* and *C. albicans* [80]. A recent in vitro study showed that the combination of curcumin and polymyxin-B displayed a synergistic effect against Gram-positive and Gram-negative bacteria [81]. 

## 7. Therapeutic Challenges and Solutions

Due to its low side effects and wide range of conventional applications, curcumin has been used in several antimicrobial studies. The intrinsic physicochemical characteristics of curcumin derivatives, such as their low bioavailability, hydrophobic nature, photo-degradation, rapid metabolism, chemical instability, and short half-life, are the major challenges that restrict their pharmaceutical impact despite their wide spectrum of results [82]. Novel strategies have recently been implemented to attempt to resolve these limitations and to boost the therapeutic ability of curcumin. These problems are being overcome by integrating curcumin in nanoformulations. Using different methods to integrate curcumin into nanocarriers is a fruitful and effective alternative for increasing curcumin’s biological function that also improves its solubility, bioavailability, long-term circulation, and retention in the body as well as overcomes curcumin’s physiological barriers. As such, nanocurcumin fabrication is a major tool and solution to the posed therapeutic challenges that are in the way of curcumin delivery. 

### 7.1. Liposomes

Liposomes are lipid-based nanocarriers that are designed in such a way that they resembles the structure of the cell membrane [83]. The relevance of incorporating therapeutic substances into liposomal gel formulations for acne treatment was illustrated in a report. The curcumin’s liposomal dispersion was transformed into a gel form using carbopol. Its co-delivery with azithromycin-loaded liposomes showed a synergistic effect compared to their individual delivery against acne bacteria (*Propionibacterium acnes*) that are sensitive and resistant to macrolide [84]. The activity of curcumin-liposomes was evaluated in chronic skin wounds and infections. The in vitro results showed that the curcumin-liposome had enhanced penetration, suggesting a high curcumin biological activity [85]. Similarly, curcumin-loaded propylene glycol liposomes were designed and showed significant antibacterial action in the treatment of second degree burns [86]. Another study showed that combination therapy using curcumin and berberine-loaded liposomes indicated a synergy between them and showed a significant effect against *S. aureus* [79].

### 7.2. Nanostructured Lipid Carriers

Curcumin and ampicillin were loaded in nanoparticles and formed into an ointment and emulgel, with both formulations an affective antibacterial action during wound healing compared to the control and animal group. No toxic effect was observed during the research study [87]. The use of a nanostructured lipid carrier is a promising delivery system for topical drugs. In this regard, a research study was conducted focusing on the treatment of *Acne vulgaris* and psoriasis using curcumin-loaded nanostructure carriers. The results indicated that the curcumin-loaded nanostructure carriers posed an enhanced permeation to the skin layers with high stability and less toxicity, findings that were confirmed through cell viability studies. The cell uptake was higher compared to free curcumin [88]. This shows that curcumin delivery through a nanostructured lipid carrier can significantly improve curcumin’s efficacy.

### 7.3. Solid Lipid Nanocarriers

Solid lipid nanoparticles can be used for both hydrophilic and hydrophobic drugs to achieve controlled drug release. Using high-pressure homogenization, curcumin-loaded solid lipid nanoparticles were fabricated. The resultant nanoparticles were highly stable and showed an enhanced antibacterial effect due to the presence of cholesterol, which caused improved curcumin penetration into the tested bacteria. In addition, the maximal loading efficacy, extended release profiles, and significant therapeutic effect were observed with the curcumin-loaded solid lipid nanoparticles [89]. Wastewater contamination caused by microbes and toxic substances is a major environmental concern. A research study on hospital wastewater containing pathogens was carried out for microbe removal. In vitro antibacterial activity was determined using curcumin-loaded solid lipid nanoparticles and as well as with free curcumin. The results of the study indicated that nanocurcumin reduced the total microbe count as well as the number of wild strains of bacteria [90]. This shows that curcumin-loaded solid lipid nanoparticles can be used as an eco-friendly tool for the eradication of harmful pathogens from wastewater.

### 7.4. Nanoemulsions

To mimic the therapeutic antibacterial effect of hydrophobic curcumin, Kole et al. designed curcumin-loaded oil-in-water nanoemulsion. A significant antimicrobial effect was observed against *E. coli* and *Bacillus subtilis*. A 0.5% curcumin nanoemulsion showed an inhibitory effect for 15 min [91]. Similarly, the same study was performed on the surfaces of medical textiles against *E. coli* and *B. subtilis*. The results showed that a curcumin-loaded nanoemulsion can be used for wound care products, and this strategy will provide more advances toward medical textile expansion [92]. To improve the water solubility of curcumin to achieve maximum therapeutic output, curcumin oil-in-water nanoemulsion was optimized and was employed against Gram-positive and negative bacteria. The results of the study indicated that the curcumin-loaded nanoemulsion was effective against Gram-positive bacteria [93]. The variation in the effects may be attributed to the differences in the cell permeability and structure as well. In addition, a curcumin nanoemulsion system was optimized using a Box–Behnken design (experimental designs for response surface methodology). The delivery system was evaluated for the treatment of *E. coli* and was administered vaginally. The results indicated that the curcumin-loaded nanoemulsion showed effective antibacterial action in the urinary tract, which was validated from the biodistribution results [94]. 

A curcumin nanoemulsion was evaluated in another study to determine its antiviral potential against dengue virus using a plaque assay and inhibitory concentration calculations. The resulting nanoemulsion exhibited high cell uptake and cytotoxicity compared to conventional curcumin solutions. The virus count was reduced significantly as well [95]. This indicates that nanoemulsions boost curcumin’s physicochemical properties, allowing them to show an enhanced antiviral effect. Due to its resistance to antifungal drugs as well as the negative influence that it has on women’s lives, vulvovaginal candidiasis has gained attention. As curcumin and piperine are hydrophobic in nature with low bioavailability, they were both combined to fabricate a nanoemulsion that was evaluated for its antifungal activity. The results of the study showed that combination therapy with the concerned nanoemulsion resulted in significant antifungal activity compared to individual therapy. Similarly, a turmeric-based oil-in-water nanoemulsion was prepared and applied to determine its antifungal activity against *Podosphaera xanthii*. The results of the study revealed that the turmeric oil nanoemulsion of turmeric inhibited fungus growth and showed a remarkable antifungal effect [96].

### 7.5. Nanocomposite Systems

Due to various potential combinatorial properties, nanocomposite systems have received a lot of attention. Since antibacterial agents may be introduced on the membrane and because the nanostructure provides stability and promotes antibacterial action, the rational manipulation of the nanostructures’ shells and cores exerts important antibacterial activity. In this regard, curcumin–silver nanocomposites were fabricated and evaluated to determine their antimicrobial action against *E. coli* and *B. subtilis*. In the presence of curcumin, silver attaches to the bacterial cell wall and produces reactive oxygen species and causes them to rupture [97]. In another study, zinc oxide nanoparticles were fabricated in such a way that the curcumin nanolayer was uniformly spread over the surface of the zinc oxide nanoparticles. The nanocomposite was evaluated against Gram-positive and Gram-negative bacteria, and the curcumin nanoparticles showed a significant inhibitory effect against the bacteria compared to amoxicillin [98].

A curcumin–polyurethane nanocomposite that led to the complete elimination of bacteria was designed by another research study and showed remarkable antibacterial action against *E. coli* [99]. In diabetic wounds, the in vitro and in vivo usefulness of a curcumin–cellulose nanocrystal composite as an antimicrobial agent was assessed. The results indicated the regeneration of hair follicles and sebaceous glands in the skin tissue [100]. Thus, this shows that curcumin nanocomposite systems can be used to treat diabetic wounds. Liu et al. developed a curcumin–chitosan nano composite system that was assessed to determine its antibacterial action against *S. aureus* and *Rhizoctonia solani.* Strong antibacterial action was observed [101]. In the same way, curcumin-loaded mesoprous silica nanoparticles were loaded into a chitosan film. This composite system was evaluated to determine its antibacterial action against *E. coli* and showed significant antibacterial action [102]. A curcumin-loaded polyvinyl pyrollidone–chitosan nanocomposite also showed outstanding antibacterial action. In addition, due to the efficient swelling of polyvinylpyrollidone posed an efficient sustained release for curcumin [103].

### 7.6. Polymeric Micelles 

In *P. aeruginosa,* the antibacterial activity of curcumin that had been encapsulated in micelle nanoparticles was assessed. It was observed that the polymeric micelles significantly suppressed the efflux pump expression in the mentioned bacteria. At the same time, the bacteria were also treated with ciprofloxacin. The results suggested that the curcumin polymeric liposomes showed remarkable antibacterial action compared to individual therapy with ciprofloxacin [104]. It can be concluded from this study that curcumin can be used as a complementary medication to help in the eradication of ciprofloxacin-resistant isolates while also increasing the antibiotic’s effectiveness by reducing the efflux pumps as well as the antibiotic retention on bacterial cells. Similarly, curcumin that had been encapsulated into pluronic polymeric micelles showed an enhanced antimicrobial effect with greater entrapment efficiency. The curcumin was determined to be stable and demonstrated greater solubility and higher antimicrobial therapeutic potential in this type of polymeric micelle system [105].

### 7.7. Polymeric Nanoparticles

The use of biodegradable polymeric nanoparticles for the delivery of phenolics has been established due to their unique features, such as their high endocytosis efficiency, biocompatibility with the system, lower levels of body clearance, and high pharmacokinetic potential [106]. Curcumin-loaded chitosan phosphate nanoparticles were developed that showed enhanced antifungal as well as enhanced antibacterial action against Gram-positive and Gram-negative bacteria. The greater and more sustained curcumin release was found more at acidic pH levels [107]. It was suggested that curcumin has photodynamic effects. This was confirmed in a research study that loaded curcumin into photosensitizer-based polymeric nanoparticles. The resultant nanoformulation was tested against bacteria. The results indicated that the particle size greatly influenced the antimicrobial phototoxicity. Curcumin alone did not reduce the bacterial growth, confirming its photodynamic effects [108]. Periprosthetic joint infections are difficult to treat with antibiotics. Curcumin-loaded polyvinylpyrrolidone nanoparticles were evaluated to determine their antimicrobial action against *S. aureus* in periprosthetic joint infections [109]. A few of the curcumin-loaded polymeric nanoparticles have been developed as well as their antibacterial effects are shown below in Table 2.

### 7.8. Hydrogels

Hydrogels are safe nanocarriers and can be employed for prolonged use [115]. Curcumin-loaded hybrid hydrogels were formulated and were assessed to determine their antibacterial action against E. coli and S. aureus. The results showed stronger antibacterial action with controlled release and a high loading efficiency [116]. In addition, thermosensitive curcumin-loaded nanohydrogels were designed and observed to determine their antimicrobial action. The results of the study suggested a brilliant antibacterial effect, with a cell death rate of 90% [117]. Another study loaded curcumin into a chitosan-g-pluronic copolymer, and this was formulated into an injectable form. The results suggested that apart from its burn wound repair capability, it also showed a good antimicrobial effect [118,119]. Similarly, curcumin-loaded cellulose–epichlorohydrin–zinc oxide hybrid hydrogels were developed and were evaluated to determine their antifungal and antibacterial effects against Trichophyton rubrum and Staphylococcus aureus. Excellent antifungal and antibacterial effects were observed with the designed curcumin hydrogels, showing their anti-infective potential against skin infections [120,121]. Another curcumin-loaded thermosensitive hydrogel was developed, and its activity was assessed in rats. Enhanced antimicrobial results were obtained from the curcumin-loaded hydrogel during the treatment of infected cutaneous wounds [122,123].

### 7.9. Miscellaneous

Nanofibers based on curcumin-loaded silica nanoparticles were designed and applied to MRSA to observe their antibacterial potential. Both the in vitro and in vivo results showed more significant antibacterial effects than pure curcumin [124,125]. A research group designed curcumin-loaded polycaprolactone–gum tragacanth nanofibers to evaluate their antimicrobial potential. The results showed that the curcumin-loaded nanofibers performed well against *S. aureus* (99.9%) [126]. Curcumin-loaded polyurethane–dextran nanofibers showed the synergistic antimicrobial potential of curcumin [127]. A combination of curcumin-loaded polymers was used to fabricate nanofibers that showed efficient bactericidal action [128]. 

Nanocrystals designed for curcumin delivery that ranged in size from 2 to 40 nm resulted in curcumin having better water diffusion. Due to the high bioavailability of the curcumin-loaded nanocrystals, which can be attributed to their reduced particle size, significant antimicrobial activity was observed [129]. Another study revealed that curcumin-loaded nanocrystals improved curcumin cellular uptake and bioavailability against *Escherichia coli*, *Streptococcus aureus,* and *Micrococcus luteus,* displaying their antimicrobial potential [130]. Quantum dots are nanocrystals with semiconductor properties. In a research study, the stability and solubility of curcumin was improved by loading it into quantum dots. The designed curcumin-loaded quantum dots showed improved antimicrobial activity [131].

## 8. Curcumin in Orthopedics: Antibacterial and Osteogenic Effects

In orthopedic implant surgery, cements are the pillars that ensure the that the implant hooks to the bone. The main risk factors of prosthetic surgery are periprosthetic infection and the implant loosening over time. Mechanical strength, biocompatibility, and antimicrobial properties are required for the durability and efficiency of orthopedic implants. Eren and his co-researchers reported on antibacterial bone fillers that were prepared for orthopedic surgery application by synthesizing polymethylmethacrylate with curcumin. The cement demonstrated adequate surface biocidal activity as well as high eukaryotic cell viability, both behaviors indicating the potential of the new material for orthopedic applications [119]. In another research study, a bioactive nanoporous magnesium–calcium silicate coating on a polyetheretherketone surface was co-loaded with curcumin and geniste, a plant-derived isoflavone with antioxidant properties. The results demonstrated that the double-loading of drugs provided the materials with good antibacterial and osteogenic activity. Additionally, indeed, the addition of curcumin and genistein to the nanoporous coating conferred high antibacterial activity against *S. aureus* and *E. coli* and promoted the in vitro proliferation and differentiation of rat bone mesenchymal stem cells, both effects being particularly desirable for orthopedic applications [121]. Very recently, Lee et al. developed a novel bioactive bone substitute with antibiofilm activity by functionalizing hydroxyapatite with curcumin. Noticeably, this new curcumin-based hydroxyapatite provided sufficient curcumin concentration flux for 14 days. The curcumin-functionalized HA inhibited *S. aureus* and *P. aeruginosa* biofilm formation and had a stronger antibiofilm effect against S. aureus compared to *P. aeruginosa*. Moreover, the curcumin-functionalized HA was non-toxic towards the human osteoblast-femoral cell line [123]. Using an electrophoretic deposition technique, Virk et al produced a multilayer coating comprising chitosan and curcumin to confer bioactive and antibacterial properties to orthopedic implants [125].

## 9. In Vitro Release Kinetics of Curcumin

Stability and solubility are the main concerns with curcumin. In this context, a curcumin-loaded ZEIN–*N*-(2-hydroxy) propyl-3-trimethylammonium chitosan chloride system was produced to cope with associated problems and to achieve sustained release kinetics. The results showed a 0.6% loading capacity and 92% encapsulation efficiency. The release behavior showed that curcumin release is best fir by the Higuchi model, and -(2-hydroxy) propyl-3-trimethylammonium chitosan chloride was found to be a suitable complex system fora sustained-release curcumin formulation [132]. In another study, polylactic acid/poly (ε-caprolactone) (PLA/PCL)-based electrospun mats were designed and loaded with curcumin, and its release behaviors were studied in vitro. Curcumin loading was maximized via the used polymer system, which showed effective cumulative release [133]. The results of another research study conveyed a new concept that curcumin-loaded chitosan microspheres linked to quantum dots is a potential candidate for use as a biocompatible carrier for the controlled drug delivery ability of curcumin. In association, non-Fickian behavior was observed during curcumin release from the carrier system [134]. Other studies showed a detailed description of the in vitro release kinetics for curcumin [133,135,136].

## 10. Conclusions and Prospects

Curcumin is a naturally occurring pigment and an active ingredient that is isolated from turmeric. It has been identified to have strong antimicrobial properties and to be made from a strong blend of antioxidant phytonutrients known as curcuminoids. It has broad-spectrum antibacterial action against a range of bacteria. Curcumin’s effectiveness against *H. pylori* and *M. tuberculosis*, alone or in combination with other existing antibiotics, is one of the most promising antibacterial findings. From both in vitro and in vivo research findings, it can be concluded that curcumin has emerged as a broad-spectrum antimicrobial agent that also has an additive effect with certain antibiotics when used as an adjuvant therapy. Despite this, the antimicrobial efficacy of curcumin was not tested in clinical studies with the intention of being used as a potential antibiotic in clinical practice. Curcumin has therapeutic potential at small concentrations, but other findings have shown that it can cause cytotoxicity. Besides its poor bioavailability, toxic effects, and inadequate solubility, curcumin often demonstrates many problems if delivered orally or intravenously because of the complexity of the human body. Curcumin has also been shown to demonstrate antifungal action against a number of fungi that are responsible for multiple infections, including dermatophytosis. It has been reported that the candidemia and candidiasis caused by *Candida* species are able to be overwhelmed by curcumin. Life-threatening diseases and infections caused by viruses were also combated by curcumin, showing its antiviral potential. In combination therapy with other phytochemicals, curcumin showed synergistic effects, and this approach was suitable for the eradication of food-borne microbes. Curcumin delivery poses many therapeutic challenges, such as low bioavailability, hydrophobicity, low body retention time, and various physiological barriers. All of these challenges lead to poor therapeutic outcomes. To overcome these challenges, the fabrication of curcumin into nanoformulations is a major solution that, in turn, results in improving its therapeutic performance.

Different infections developing resistance to a range of antibiotics is a serious concern all over the world. Because of this, curcumin’s novel bioactivity as well as recent advances in the field of nanotechnology generally, and, specifically, the development of curcumin-based nanoparticles have prompted researchers to look for new and potentially effective therapeutic agents to combat pathogenic microbes with minimal side effects and high therapeutic outcomes. The use of nano-based platforms (specialised systems for the transport of chemotherapeutic active medicines comprising colloidal nanoparticles that are submicrons in size (usually 500 nm) with a high surface to volume ratio) will not only solve the issues with curcumin but will also break down certain other barriers that are in its way. Over the last few decades, a lot of research has been carried out to evaluate various metal nanoparticles to determine their bioactivity. Similarly, while there are numerous publications on the use of curcumin in biomedicine, there is little literature on curcumin and its concerned nanoparticles. Fortunately, several researchers have recently concentrated on curcumin-based nanoparticles that can be used as an efficient and economically feasible antimicrobial agent with minimal to no side effects due to the novel bioactive properties of curcumin. To date, studies show that curcumin-loaded nanoparticles have promising bioactivity against a variety of pathogens, including those with multidrug resistance. Just a few articles, however, have mentioned their cytotoxic effects. It is completely obvious that curcumin toxicity is a complicated problem due to contradictory results from previous cytotoxicity studies. In several experiments, curcumin has been shown to have cytotoxic properties above specific doses. As a result, the toxicological issues around curcumin-loaded nanoparticles must be thoroughly investigated. In light of these findings, it is expected that the use of curcumin-based nanoparticles would be beneficial for the treatment of microbial pathogens. 

Among big challenges, the poor bioavailability of curcumin due to its hydrophobic nature and poor stability is a major challenge in its clinical applications. Curcumin faces extensive fast liver metabolism and hence quick systemic elimination, which is another challenge for curcumin delivery in clinical a set up. In association, curcumin’s short half-life and low drug efficacy are other challenging hurdles that need to be overcome. To tackle these issues, nanotechnology scaffolds are the best-explored technology that are currently under investigation need more exploration in future to overcome the challenges facing curcumin. It is recommended that the nanocurcumin’s therapeutic capabilities against infectious diseases be investigated. Nanocurcumin has to be tested in clinical trials before use as an adjunct drug for routine care. In general, with the rise in antibiotic resistance, nanocurcumin holds promise in overcoming drug resistance due to its limited side effects. Although nanocurcumin has antimicrobial properties against pathogens by inhibiting certain key molecules that are involved in their survival and growth, the regulation of the host’s immunomodulation processes may be associated with it. Therefore, the assessment of nanocurcumin’s effectiveness in humans during infection with an infection disease is required. Potential developments based on the exciting bioactivities of curcumin and curcumin-loaded nanoparticles are anticipated in the coming years. Curcumin-loaded nanoparticles will be the subject of study around the world, with the goal of developing successful broad-spectrum therapeutic molecules for the treatment of pathogenic microbes and for overcoming the associated challenges. Aside from that, further research into the toxicity of curcumin and its nanoparticles is needed to determine whether they have any harmful effects to humans or the environment. Keeping all of these aspects in view and their consequent control could make curcumin an effective next-generation antimicrobial candidate.

## Figures and Tables

**Table 1:** An overview of the experimental studies on the antibacterial effects of curcumin.

Microorganism (Bacteria)	Experimental Status	MIC (µg/mL)	Research Findings	References
*Streptococcus mutants*	In vitro study	128	Curcumin inhibited the adherence of microbes to the periodontal cavity	[29]
*Staphylococcus aureus*, *Escherichia coli*, *Enterococcus faecalis, Pseudomonas aeruginosa*	In vitro study	25	Curcumin inhibited the growth of all of the tested bacteria via rupturing their cell membranes and showed potent antibacterial activity	[18]
Methicillin-resistant *Staphylococcus aureus*	In vitro study	125	Curcumin lowered the MICs of oxacillin, ciprofloxacin, norfloxacin, and ampicillin against MRSA	[19]
*Escherichia coli*	In vitro study	12	Curcumin has potent inhibitory activity against *E. coli*	[30]
*Klebsiella pneumonia*, *Bacillus subtilis*, *Enterobacter aerogenes*, *E. coli, S. aureus*, *Proteus mirabilis*, *P. aeruginosa*	In vitro study	34	Curcumin showed potent antibacterial activity compared to demethoxycurcumin and bisdemethoxycurcumin	[31]
*Escherichia coli*	In vitro study	8	Curcumin prevented the SOS reaction of *E. coli,* initiated via levo-floxacin	[32]
*P. aeruginosa*	In vitro study	8–512	The combination therapy of curcumin with azithromycin and gentamicin showed a marked synergistic antibacterial effect	[33]
*Staphylococcus aureus*	In vivo and in vitro study	2–16	Mice infected with *S. aureus* were cured with curcumin	[34]
*Salmonella typhimurium*, *Salmonella typhi*	In vivo and in vitro study	0.5–2	Curcumin showed potent antibacterial activity in a mice model	[35]
*Helicobacter pylori*	In vivo and in vitro study	5–50	Curcumin completely eradication the *H. pylori* that induced stomach injury in mice	[20]

**Table 2:** Curcumin-loaded polymeric nanoparticles and their antibacterial potential.

Source	Polymeric System	Status	Bacteria	Results	References
Synthetic polymers	Pectin-assisted curcumin-loaded polylactic acid nanoparticles	In vitro	*S. aureus* and *E. coli*	The fabricated curcumin-loaded polymeric nanoparticles displayed a strong antibacterial effect	[110]
	Curcumin-loaded polylactic acid nanoparticles	In vitro	*S. mutans*	Curcumin-loaded nanoparticles showed highwater solubility and photodynamic antimicrobial activity	[111]
Natural polymers	Curcumin-encapsulated gelatin nanoparticles	In vitro	*L. monocytogenes*, *E. coli*, *S. aureus*	Curcumin solubility was increased 39-fold and reduced the bacterial population	[112]
	Curcumin-loaded chitosan/tetra methyl orthosilicate nanoparticles	In vitro	*P. aeruginosa*	A 60% reduction in bacteria growth was observed with the application of polymeric nanoparticles	[113]
	Chitosan–carboxymethyl cellulose-based curcumin-loaded nanoparticles	In vitro	*P. aeruginosa*	Strong antibacterial action	[114]
